# Optimizing myasthenia gravis management in practice: integrating clinical scales and classification systems

**DOI:** 10.1055/s-0046-1817031

**Published:** 2026-06-25

**Authors:** José Pedro Soares Baima, Caio César Diniz Disserol, Eduardo Braga de Oliveira, Natália Merten Athayde, Lara Albuquerque de Brito, Hermany Capistrano Freitas, Cleonisio Leite Rodrigues

**Affiliations:** 1 Hospital Geral de Fortaleza, Serviço de Neurologia, Fortaleza CE, Brazil.; 2 Universidade Federal do Ceará, Hospital Universitário Walter Cantídio, Fortaleza CE, Brazil.; 3 Universidade Federal do Paraná, Hospital de Clínicas, Curitiba PR, Brazil.; 4 Instituto de Neurologia de Curitiba, Curitiba PR, Brazil.; 5 Hospital Israelita Albert Einstein, Instituto do Cérebro, São Paulo SP, Brazil.

**Keywords:** Myasthenia Gravis, Neuromuscular Junction, Patient Reported Outcome Measures

## Abstract

Myasthenia gravis is an antibody-mediated disorder of the neuromuscular junction. Fatigability and fluctuations are hallmarks of the disease, posing a challenge: evaluation through a single physical examination may not reflect disease control. A shift towards patient-reported Outcomes is a new paradigm in the current practice, and these outcomes are usually assessed through validated scales. In the present paper, we discuss some of the most used scales in the clinical practice, such as the Myasthenia Gravis Foundation of America (MGFA) scale, the Myasthenia Gravis Activities of Daily Living (MG-ADL) scale, and the 15-Item Myasthenia Gravis Quality of Life (MG-QOL15) scale. Definitions of remission, minimal manifestation, high-burden disease, highly-active disease, and others are reviewed, and correlations involving these definitions and the scales are presented. Guidance on how to assess disease severity, therapeutic response, and a rationale for treatment escalation are explored.

## CLINICAL VIGNETTE

**Figure 1:**
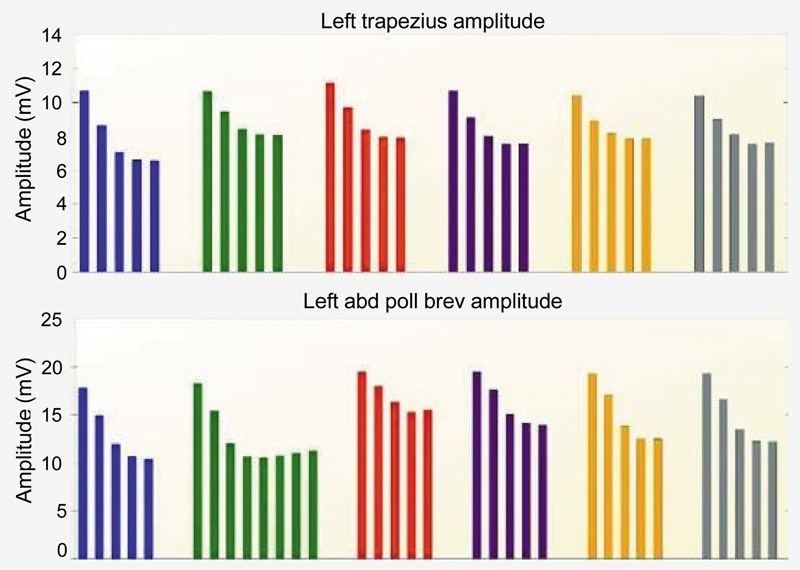
Repetitive stimulation with low frequency shows motor amplitude decrements of around 40% in 2 muscles with post-effort facilitation followed by exhaustion.

A 42-year-old male presented with a 3-year history of generalized weakness and bulbar symptoms. Based on the clinical features, autoimmune myasthenia gravis (MG) was suspected. Upon presentation, the disease was classified as IIIB according to the Myasthenia Gravis Foundation of America (MGFA) scale. The score on the Myasthenia Gravis Activities of Daily Living (MG-ADL) scale was 10, and the score on the 15-Item Myasthenia Gravis Quality of Life (MG-QOL15) scale was 45. The physical examination was remarkable for binocular diplopia on primary gaze and unilateral ptosis in sustained upward gaze (Simpson's test), associated with mild facial weakness. Mild proximal motor weakness was also perceived, which was classified as grade 4 on the Muscle Research Council (MRC) scale. After a prolonged time of consultation, the patient developed moderate dysarthria. Repetitive nerve stimulation showed a decrease of more than 10% in more than 2 muscles, consistent with postsynaptic neuromuscular junction dysfunction ( Figure 1 ). The anti-acetylcholine receptor antibody (Anti-AChR) was positive in high titers. A chest computed tomography (CT) scan was unremarkable. Treatment was initiated with prednisone, pyridostigmine, calcium, and vitamin D. Azathioprine was started as an immunosuppressant and a steroid-sparing agent. This drug was increased until reaching the target dose of 2.5 mg/kg. After 1 year of treatment, the MG-ADL score was 8, and the Mg-QOL 15 score, 41. How should this case be managed?

## FROM PRESENTATION TO RESOLUTION: LESSONS LEARNED

### How to objectively evaluate a patient with MG through scales

Myasthenia gravis is an antibody-mediated disorder of the neuromuscular junction. Fatigability and fluctuation are hallmarks of the disease. 1 This poses a unique challenge: patients may report multiple symptoms while presenting with fewer signs during the neurological evaluation. 2 To overcome this issue and improve patient assessment, consideration of patient-reported outcomes (PROs) has increased. 2 More than just study outcomes, PROs are currently incorporated in the clinical practice through validated scales. 3

Clinically, the degree of weakness can be classified as *ocular* or *generalized* (mild, moderate, or severe), with or without predominantly-bulbar symptoms. 4 These characteristics are summarized in the MGFA scale. The initial classification should be performed upon presentation, and it should be periodically reassessed to monitor and grade disease severity, also known as *MGFA postintervention status* . 4 However, the disadvantages of this scale include its subjectivity and the lack of quantification regarding the impact on daily living.

The MG-ADL is an 8-item patient-reported scale, which is linearly scored from 0 to 3, with a maximum total score of 24. 3 The questions include domains of speech, chewing and swallowing, shortness of breath, and vision, among others. The higher the score, the more limited the activities of daily living. The instrument is useful in the clinical practice and as an outcome measure in clinical trials. 3 A two-point variation is considered a clinically-significant change, and it reflects clinical improvement or worsening symptoms, with the best balance between sensitivity and specificity. 2 5

Another scale recommended in the clinical practice is the MG-QOL15, which is intended to reflect the effectiveness of the interventions and the quality of life due to myasthenic symptoms. 2 It comprises a 15-item questionnaire covering domains of mobility, symptoms, contentment, and emotional well-being. 6 The items are scored from 0 to 4, with a maximum total score of 60, or a revised version that scores each item from 0 to 2, with a maximum of 30. 6 Higher scores indicate poorer quality of life, and a reduction of more than 7 points in the 60-point scoring system correlates with clinical improvement. 6

There are moderate-to-high correlations among the scales presented here, including correlations with physician-based complementary assessment tools, and these scales are now considered the standard of care for the assessment of patients with myasthenia gravis. 2 3 4 6

There have been some efforts to translate certain scales to Brazilian Portuguese: the Quantitative Myasthenia Gravis Scale (QMG), the Myasthenia Gravis Composite Score (MGC), and the MG-QOL-15. 7 8 9 The QMG and MGC are physical examination-based, take longer to administer or need complementary tools (QMG), and are used especially in research settings or situations in which the MGADL may be inconsistent with the clinical status. 2 As previously highlighted, there is also a shift toward PROs and less reliance on the isolated examination due to the characteristic fluctuation of the disease. 2

### What is a therapeutic response, and what is the goal of treatment?

Originally developed as a study outcome, the MGFA has defined two major therapeutic goals: remission and minimal manifestation status. Complete remission is defined as 1 year or longer without signs and symptoms and without the use of pyridostigmine. 6 If pyridostigmine or immunosuppressants are still required to remain without signs and symptoms, the condition is referred to as *pharmacological remission* . 6 Minimal manifestation status, in turn, is defined as minimal signs and symptoms even during the pyridostigmine treatment. 6

More recently, a shift from physician-based scores to patient preferences has emerged: the PASS in MG. A single yes or no question has been validated as a goal to evaluate control in myasthenia: 10 “Considering all the ways you are affected by myasthenia, if you had to stay in your current state for the next months, would you say that your current disease status is satisfactory?”. 10 This query has valued patient wellness over just “feeling better than before”. 10

The PASS correlates with established scales, corresponding to MG-ADL score ≤ 2 and MG-QOL-15 score ≤ 8. 10 11 The MG-ADL score ≤ 2 has also been labeled *minimal symptom expression* (MSE) 3 ( Table 1 ).

**Table 1:** Definitions of major goals in myasthenia gravis

Status	Signs and symptoms	MG-ADL score	Medication
Complete **stable** remission	None **for 1 year**	0	None
Pharmacological remission	None **for 1 year**	0	Pyridostigmine **and /or imunossupressants**
Minimal symptom expression	Minimal	0–2	None or pyridostigmine*

Abbreviation: MG-ADL, Myasthenia Gravis Activities of Daily Living scale.

Note: *Some authors consider a low dose of steroids (lower than 5 mg prednisone) acceptable.

Although the ideal goal is remission in all patients, this is usually unrealistic due to treatment-related adverse effects. The most reasonable goal is to achieve a positive PASS and an MG-ADL compatible with MSE, ideally with no therapy or with the lowest dose of symptomatic therapy. 2 3 4 6 12 ( Table 2 ).

**Table 2:** Summary of goals as defined in the scales most used in the clinical practice

Scale	Well-controlled	Partially-controlled	Poorly-controlled
MGFA	NA	IIA	> IIA
MG-ADL	0–2	3–5	≥ 6
MG-QOL	< 8	> 8	NA
PASS	Yes	No	No

Abbreviations: MG-ADL, Myasthenia Gravis Activities of Daily Living scale; MGFA, Myasthenia Gravis Foundation of America scale; MG-QOL15, 15-Item Myasthenia Gravis Quality of Life; NA, not applicable; PASS, Patient-Acceptable Symptom State.

Note: There are correlations involving the scales and patient satisfaction, as there are also correlations with other scales, such as the Quantitative Myasthenia Gravis Scale (QMG) and the Myasthenia Gravis Composite Score (MGC).

A key secondary endpoint to keep in mind is to reach the lowest effective dose of steroids in the shortest possible time to minimize side effects. 2 12 An acceptable dose is lower than 5 mg of prednisone to MSE.

Ensuring patient safety is of paramount importance, and it should remain a constant objective throughout all stages of disease management. Prioritizing safety helps to prevent severe complications, particularly in the first 2 years of the disease. Complications can be driven by exposure to viral pathogens or by treatment failure. 13 For every 1-point increase in the MG-ADL score, there is a 13% increase in the number of exacerbations within the previous 6 months. 14

### When to consider a highly-active disease and clinical refractoriness?

Recently, the definition of highly-active disease was incorporated into international guidelines, and we recommend further reading of the article by Wiendl et al. 4 It is generally defined as MGFA greater than IIA over 2 years or over 1 year with severe exacerbations/myasthenic crises, despite adequate disease-modifying and symptomatic treatment. 4

Refractory disease is a broader clinical term with various definitions. It comprises ongoing symptoms with failure to respond to immunotherapy of sufficient dose and duration, severe side effects leading to discontinuation of therapy or the need for rescue medications to improve symptoms. 4 The occurrence of a myasthenic crisis, or impending myasthenic crises, despite adequate disease-modifying treatment, is regarded as *refractory disease* . This concept overlaps with that of highly-active disease. 4

In addition to highly-active and refractory disease, the concept of high-burden disease is also used, and it is associated with MG-ADL score ≥ 6. 12 A high-burden disease led to an escalation of therapy in most patients in the MG patient registry. 12 Patients with MG and high symptom burden at baseline have a low probability of achieving MSE after 1 year, even with therapeutic adjustments. 12

### Is there any option for highly-active or refractory cases?

It is worth mentioning that all refractory cases should have their diagnosis reassessed. Many of these patients have alternative diagnoses. 1 This was not the case of the patient in the clinical vignette: he had neurophysiological evidence of neuromuscular junction impairment and had a high-titer positivity for anti-AChR.

Aside from classic medications (pyridostigmine and non-steroid immunosuppressants), there are some medications approved for high-burden disease or refractory cases. 4 The options include anti-CD20 monoclonal antibody (rituximab), complement inhibitors (ravulizumab), and neonatal fragment crystallizable (Fc) receptor inhibitors (efgartigmod, rozanolixizumab, and nipocalimab). The prescription of some of these therapies also depends on the anti-AChR status. Currently, not all of these options are available in Brazil or have a label indication for MG.

In addition to pharmacological treatment, thymectomy is a well-established strategy to improve long-term remission rates. The procedure should be considered for all patients with anti-AChR-positive generalized myasthenia gravis aged 18 to 65 years, preferably within the first 5 years after disease onset. 4

**Figure 2:**
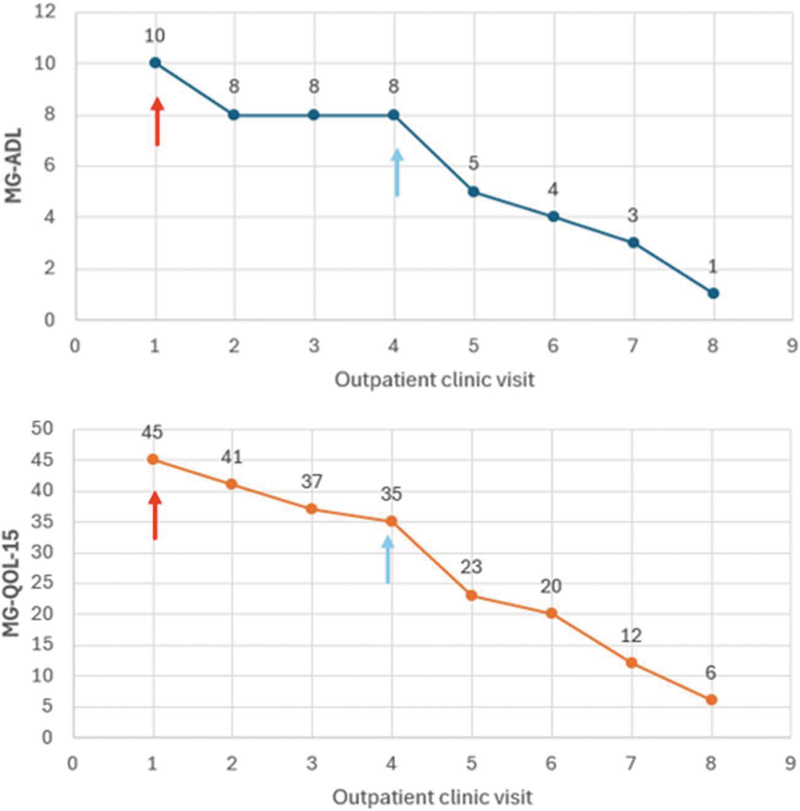
Evolution of the patient's scores on the Myasthenia Gravis Activities of Daily Living (MG-ADL; left panel) scale and the 15-Item Myasthenia Gravis Quality of Life (MG-QOL15; right panel) scal. Each point represents an outpatient clinic visit. The orange arrow represents the initial treatment, and the light blue arrow represents rituximab.

The patient in the clinical vignette started on steroids and azathioprine, as these are first-line medications according to some guidelines and due to drug availability in the Brazilian public health system. The patient was referred to thymectomy but declined the surgical procedure. He persisted all the time with MG-ADL scores above 5 and MGFA grade above IIB for another year, compatible with a high-burden, severe refractory MG. After ensuring complete vaccination, rituximab was initiated and the corticosteroids were discontinued, and control was achieved over 1 year. Rituximab is an off-label medication in Brazil, but it was chosen due to local hospital availability.. At the last follow-up visit, the patient's MGFA was I, the MG-ADL score was 1, and the MG-QOL1-5 score, 6. A summary of the evolution of the scales is shown in Figure 2 . The patient complained of rare episodes of diplopia. The physical examination was unremarkable. The case herein reported illustrates how validated scales can be integrated into the clinical practice to assess severity and therapeutic response and to provide a rationale for treatment escalation in refractory MG.

## Notes

TEACHING POINTS

1. Assessment through scales better represents disease activity and control than a single office evaluation.
2. The goal of treatment is based on a patient's feeling of wellness, reflected in the Patient-Acceptable Symptom State (PASS), which correlated with scores on the scales.
3. The new concepts of highly-active and high-burden disease may guide escalation in therapy: both definitions are based on the assessment by the scales herein presented.
